# Molecular Epidemiology of SARS-CoV-2 during Five COVID-19 Waves and the Significance of Low-Frequency Lineages

**DOI:** 10.3390/v15051194

**Published:** 2023-05-18

**Authors:** Kathleen Subramoney, Nkhensani Mtileni, Jennifer Giandhari, Yeshnee Naidoo, Yajna Ramphal, Sureshnee Pillay, Upasana Ramphal, Akhil Maharaj, Derek Tshiabuila, Houriiyah Tegally, Eduan Wilkinson, Tulio de Oliveira, Burtram C. Fielding, Florette K. Treurnicht

**Affiliations:** 1 School of Pathology, Faculty of Health Sciences, University of the Witwatersrand, Johannesburg 2193, South Africa; florette.treurnicht@nhls.ac.za; 2 Department of Virology, National Health Laboratory Service, Charlotte Maxeke Johannesburg Academic Hospital, Johannesburg 2193, South Africa; nkhensani.mtileni@nhls.ac.za; 3 Centre for Vaccines and Immunology, National Institute for Communicable Diseases, Johannesburg 2131, South Africa; 4 KwaZulu-Natal Research Innovation and Sequencing Platform (KRISP), Nelson R Mandela School of Medicine, University of KwaZulu-Natal, Durban 4001, South Africa; jennifer.giandhari@gmail.com (J.G.); yajna.ramphal@gmail.com (Y.R.); pillaysureshnee@gmail.com (S.P.); upasanaramphal@gmail.com (U.R.); akhilmaharaj@gmail.com (A.M.); houriiyah.tegally@gmail.com (H.T.); tuliodna@gmail.com (T.d.O.); 5 Centre for Epidemic Response and Innovation (CERI), School of Data Science and Computational Thinking, Stellenbosch University, Stellenbosch 7600, South Africa; yeshnee.m@gmail.com (Y.N.); derektshiabuila@gmail.com (D.T.); ewilkinson@sun.ac.za (E.W.); 6 Molecular Biology and Virology Research Laboratory, Department of Medical BioSciences, University of the Western Cape, Cape Town 7535, South Africa; bfielding@uwc.ac.za

**Keywords:** SARS-CoV-2, low frequency, lineages, molecular epidemiology

## Abstract

SARS-CoV-2 lineages and variants of concern (VOC) have gained more efficient transmission and immune evasion properties with time. We describe the circulation of VOCs in South Africa and the potential role of low-frequency lineages on the emergence of future lineages. Whole genome sequencing was performed on SARS-CoV-2 samples from South Africa. Sequences were analysed with Nextstrain pangolin tools and Stanford University Coronavirus Antiviral & Resistance Database. In 2020, 24 lineages were detected, with B.1 (3%; 8/278), B.1.1 (16%; 45/278), B.1.1.348 (3%; 8/278), B.1.1.52 (5%; 13/278), C.1 (13%; 37/278) and C.2 (2%; 6/278) circulating during the first wave. Beta emerged late in 2020, dominating the second wave of infection. B.1 and B.1.1 continued to circulate at low frequencies in 2021 and B.1.1 re-emerged in 2022. Beta was outcompeted by Delta in 2021, which was thereafter outcompeted by Omicron sub-lineages during the 4th and 5th waves in 2022. Several significant mutations identified in VOCs were also detected in low-frequency lineages, including S68F (E protein); I82T (M protein); P13L, R203K and G204R/K (N protein); R126S (ORF3a); P323L (RdRp); and N501Y, E484K, D614G, H655Y and N679K (S protein). Low-frequency variants, together with VOCs circulating, may lead to convergence and the emergence of future lineages that may increase transmissibility, infectivity and escape vaccine-induced or natural host immunity.

## 1. Introduction

Severe acute respiratory syndrome coronavirus 2 (SARS-CoV-2) is continually evolving, resulting in the emergence of several lineages and variants of concern (VOC). The first VOC detected was Alpha (B.1.1.7) which originated in the UK [1], followed by Beta (B.1.351), which originated in South Africa (SA) in August 2020 [2,3]. Delta (B.1.617.2) was initially detected in India in April 2021 but spread rapidly to other countries, including SA, and led to the emergence of several highly transmissible sub-lineages [4]. The most recent VOC identified was Omicron (B.1.1.529), which is divided into BA.1, BA.2, BA.4 and BA.5 lineages and several sub-lineages [5]. Omicron BA.1 initially circulated in November 2021 and was replaced by BA.2 in early 2022, from which BA.4 and BA.5 emerged by April 2022. Currently, Omicron BA.5 and its sub-lineages are driving the SARS-CoV-2 infection globally [1]. In SA, the National Department of Health implemented stringent preventative measures, including wearing masks, social distancing and different levels of national lock-down with each COVID-19 wave [6]. Longer-term prevention strategies included the rollout of vaccines: five vaccines have been approved for use in SA, including Covishield (Oxford/AstraZeneca), Comirnaty (Pfizer/BioNTech), Janssen (Johnson & Johnsons), COVID-19 vaccine MC Pharma (MC Pharma), and Coronavac (Sinovac) [7].

Since May 2023, Africa has confirmed 9,525,097 SARS-CoV-2 cases and 161,630 genomes sequenced, while South Africa had ~4 million confirmed cases, of which 52,720 genomes were sequenced [8,9]. Prior to the circulation of Beta in SA, from March to August 2020, 16 different SARS-CoV-2 lineages were detected in SA, of which B.1.1.54, B.1.1.56 and C.1 drove the first wave of infection [10]. The first significant mutation D614G, in the spike (S) protein, first emerged in the original B.1 lineage and subsequently became prevalent in other lineages and VOCs of SARS-CoV-2. B.1.1.54 and B.1.1.56 are sub-lineages of the B.1 lineage that have accumulated additional mutations, resulting in approximately 14 mutations compared to the original Wuhan-Hu1 strain. The C.1 lineage is a separate variant of the virus that was identified with 16 mutations compared to the Wuhan-Hu-1 strain. By mid-September 2020, SA saw approximately 42 different SARS-CoV-2 lineages, with at least 24 lineages circulating within an epiweek during peak infections [10]. C.1.1 became the dominant lineage with S protein mutations S477N, A688S and M1237I and with additional mutations similar to B.1.525 (Q52R and A67V), a globally circulating lineage [3]. Convergence was observed between the C.1.2 lineage and other VOCs, but this lineage did not dominate in SA or globally due to its sensitivity to neutralising convalescent plasma antibodies from Beta- and Delta-infected persons [11].

The rapid emergence and circulation of novel lineages were not unexpected, as previous reports described the rate of mutations being 0.8–2.4 × 10^−3^ nucleotide substitutions/site/year [12]. Such rapid evolution allows the virus to adapt to environmental pressures (i.e., a new host) and to evade vaccine-induced or infection-induced immunity. As VOCs evolved from the Wuhan-Hu-1 strain, SARS-CoV-2 experienced multiple convergent episodes, which enhanced infectivity and disease transmission and improved resistance mechanisms for immune escape [1]. Of note, the novel lineages are more likely to evolve from previous VOCs, as observed with Delta and Omicron [1]. Limited reports of the circulation, or re-emergence, of the earlier and less frequent lineages during the subsequent COVID-19 waves are available. To better understand the molecular epidemiology of SARS-CoV-2 lineages in SA, we described the circulation of SARS-CoV-2 lineages and VOCs detected between 2020 to 2022 in Gauteng Province, the potential significance of low-frequency lineages and their contribution to the emergence of new lineages.

## 2. Materials and Methods

### 2.1. Study Population and Samples

SARS-CoV-2 respiratory specimens from individuals of all ages from 30 March 2020 to 13 October 2022 were included in this study. The samples were tested for SARS-CoV-2 at the National Health Laboratory Service (NHLS) at Charlotte Maxeke Johannesburg Academic Hospital, Gauteng, South Africa. The samples received were from the Gauteng province, as well as referrals from the Eastern Cape, KwaZulu-Natal, Limpopo and Western Cape provinces of South Africa. This study contributed to the national surveillance for SARS-CoV-2 in South Africa, for which formal patient consent was not required. The study was approved by the University of the Witwatersrand Human Research Ethics Committee (M210119), and all participants’ data were anonymised and presented in aggregated form.

### 2.2. Next-Generation Sequencing of SARS-CoV-2 Strains

Samples with a Ct-value <31 were randomly selected, with 50–80 positive samples selected per week during peak and off-peak periods; if <50 positives were detected, all samples were sequenced. All samples selected for sequencing were submitted to the KwaZulu-Natal Research Innovation and Sequencing Platform (KRISP) for sequencing or were sequenced in-house at the NHLS Virology Laboratory. Complementary DNA was synthesised from total nucleic acids and amplified using the ARTIC protocols [2,13,14]. Nextera DNA library preparation kits (Illumina, San Diego, CA, USA) were used for sequencing with the MiSeq platform (Illumina, San Diego, CA, USA) and Nanopore ligation sequencing kits (Oxford Nanopore Technologies, Oxford, UK) for sequencing with the MinION (Oxford Nanopore Technologies, UK) [5,15]. Genome assembly was performed using genome detective [16] or Exatype [17] online tools. The Wuhan-Hu-1 strain (NC_045512.2; https://www.ncbi.nlm.nih.gov/nuccore/1798174254/, accessed on 3 April 2023) was used as the reference sequence throughout the assembly and analysis steps.

### 2.3. Data Analysis

Descriptive statistical analysis was conducted across different age groups (<5, 5–14, 15–24, 25–44, 45–60, and >60 years), gender, provinces and patient status. Consensus sequences were analysed using Nextstrain (https://clades.nextstrain.org, accessed on 20 February 2023) and pangolin (https://pangolin.cog-uk.io/, accessed on 20 February 2023) online tools for variant assignment. Nextstrain custom build time-trees were run using our custom sequence data and metadata. S protein mutations were analysed using the Stanford University Coronavirus Antiviral & Resistance Database tool (https://covdb.stanford.edu/sierra/sars2/by-sequences/, accessed on 23 February 2023).

## 3. Results

### 3.1. Study Population Demographics

A total of 2547/3797 (67.1%) SARS-CoV-2 whole genomes were successfully sequenced, with 10.9% (278/2547) from 30 March to 31 December 2020, 54.9% (1399/2547) from 1 January to 31 December 2021, and 34.2% (870/2547) from 1 January to 13 October 2022 (Table 1). From 2020 to 2022, SARS-CoV-2-positive individuals were detected across all age groups. The majority of SARS-CoV-2 infected individuals were among adults aged 25–44 years of age (47.3%; 1162/2547), with similar detection rates observed in 2020 (50.7%; 141/1278), 2021 (43.7%; 611/1399) and 2022 (47.1%; 410/870). The SARS-CoV-2 detection rate observed for females was 60.1% (1531/2547), while for males, it was 37.5% (956/2547) across the study period. Among our study, individuals residing in Gauteng Province represented 97.0% (2472/2547) of samples predominantly from the City of Johannesburg Metro district. Three percent (68/2547) included samples referred from the Eastern Cape, KwaZulu-Natal, Limpopo and Western Cape for testing at the NHLS Virology laboratory in Gauteng Province. SARS-CoV-2 positive individuals were predominant among those that sought community screening and test services (71.8%; 1828/2547), followed by out-patients (59.3%; 292/492) which were comprised of patients seen at casualty (19.3%; 492/2547), healthcare workers (16.3%; 80/492), and antiretroviral (ARV) clinic attendees (22.0%; 108/492).

### 3.2. Epidemiology of SARS-CoV-2 Lineages and VOCs, 2020 to 2022

**Figure 1:**
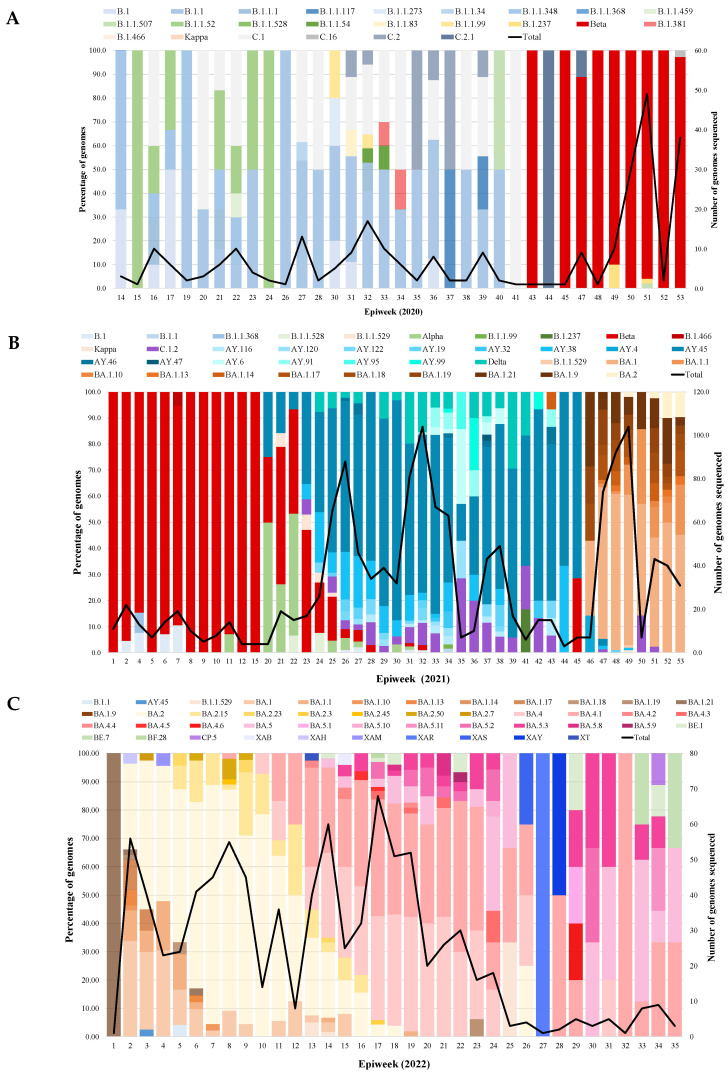
Prevalence of SARS-CoV-2 lineages over time from 2020 to 2022 by epiweek. The bar graph represents the SAR-CoV-2 lineages and VOCs identified in our study cohort. The black line graph represents the total number of samples that were sequenced during each epiweek. (**A**) Distribution of SARS-CoV-2 lineages in 2020, (**B**) Distribution of SARS-CoV-2 lineages in 2021, and (**C**) Distribution of SARS-CoV-2 lineages in 2022.

During the first COVID-19 wave, 24 SARS-CoV-2 lineages were identified among the 278 genomes sequenced in 2020 from epiweeks 14 to 53. The B.1.1 (16%; 45/278), B.1.1.52 (5%; 13/278) and C.1 (13%; 37/278) lineages were the most prevalent in 2020. Lineages present at frequencies below 5% in the same period included B.1 (3%; 8/278), B.1.1.348 (3%; 8/278), B.1.1.1 (2%; 5/278) and C.2 (2%; 6/278) (Figure 1A).

In 2020, B.1.1.52 was most prevalent from weeks 15 to 24, with the detection rate decreasing from 100% (1/1) to 33.3% (2/6) (Figure 1A). B.1.1 was observed from epiweeks 19 to 40, with detection rates fluctuating between 33.3% (2/6) to 53.8% (7/13) from weeks 27 to 40. C.1 was dominant from week 16 to 22 and 27 to 41 in 2020, with detection rates ranging from 16.7% to 100%, respectively, while C.2 remained lower at week 31 (11.1%; 1/9) to 32 (5.9%; 1/17) and 35 (50.0%; 1/2) to 39 (11.1%; 1/9). For lineages detected at lower frequencies, B.1 was scattered from week 14 to 17 (33.3% (1/3) to 50.0% (3/6)), week 30 (20.0%; 1/5), week 31 (11.1%; 1/9) and 36 (12.5%; 1/8); B.1.1.348 was only observed in 2020, from weeks 14 to 22 with detection rate of 66.7% (2/3) down to 20.0% (1/10). Beta (B.1.351) outcompeted all lineages and became dominant from epiweek 43 in 2020, driving the second wave of infection up to week 15 of 2021.

In 2021, Beta retained dominance up to week 15 at 84.2% (16/19) to 100% (4/4) and decreased to 2.9% (1/34) by week 28 (Figure 1B). Alpha (B.1.17) was also observed during the second wave, with peak detection rates at weeks 20 (50.0%; 2/4) and 22 (46.7%; 7/15) coinciding with Beta and Delta AY.46 (Figure 1B). Delta (B.1.617.2) and its sub-lineages outcompeted Beta and Alpha to dominate the third wave. Delta had an overall prevalence of 52.9% (737/1399) in 2021 (Figure 1B). Delta AY.45 sub-lineage (36.1%; 505/1399) was predominant from week 26 (58.0%; 51/88) to week 42 (73.3%; 11/15) and decreased to 2.7% (11/74) by week 47. Subsequently, Omicron BA.1 emerged and outcompeted Delta, dominating the beginning of the fourth wave (week 46 to 53) in 2021, with an overall prevalence of 30.2% (422/1399). From weeks 46 to 53, Omicron BA.1 peaked at 59.8% (55/92), followed by BA.1.18, BA.1.19 and BA.1.21 sub-lineages (Figure 1B). Omicron BA.2 started to emerge at week 49 in 2021 and continued to co-circulate with BA.1 until the end of 2021. Low-frequency lineages observed in 2021 included re-emerging B.1 and B.1.1. B.1, observed from weeks 1 to 7, with detection rates down to 10.5% (2/19) and decreasing to 0.4% from weeks 26 to 32 (Figure 1B). B.1.1 was observed at week 34 (50.0%; 1/2). C.1.2 co-circulated with Beta and Delta (Figure 1B), with an overall prevalence of 6.1% (53/870), but was observed from epiweeks 23 to 51 at very low numbers with detection rates ranging from 2.2% (1/46) to 28.6% (2/7).

In 2022, Omicron BA.2 outcompeted BA.1 from week 3, continuing through the fourth wave (Figure 1C). BA.2 retained a prevalence >50% from weeks 3 to 11, peaking in week 7 (84.4%; 38/45). Omicron BA.4 and BA.5 dominated the fifth wave of infections from weeks 13 to 42 in 2022. Omicron BA.4 and BA.4.1 were dominant at similar frequencies from weeks 13 to 26 (15.0% to 53.3%). BA.5 slowly emerged from week 23 but only dominated from week 30 (33.3%; 1/3) to week 42 (33.3%; 1/3), when positivity rates were declining. Subsequently, Omicron sub-lineages continued to circulate, and recombinant strains were identified at an overall prevalence of 2.4% (21/870). BE.1 (0.9%; 8/870) and BE.7 (0.3%; 3/870) recombinants were the most prevalent at 1.5% to 25% but scantily distributed from weeks 14 to 42. Recombinants BF.28, CP.5, XAB, XAM, XAR, XAS, XAY and XT were only identified once from weeks 4 to 42 (Figure 1C). In addition, B.1.1 was observed again at week 5 in 2022 in a single case.

### 3.3. Phylogenetic Description of VOCs Overtime

**Figure 2:**
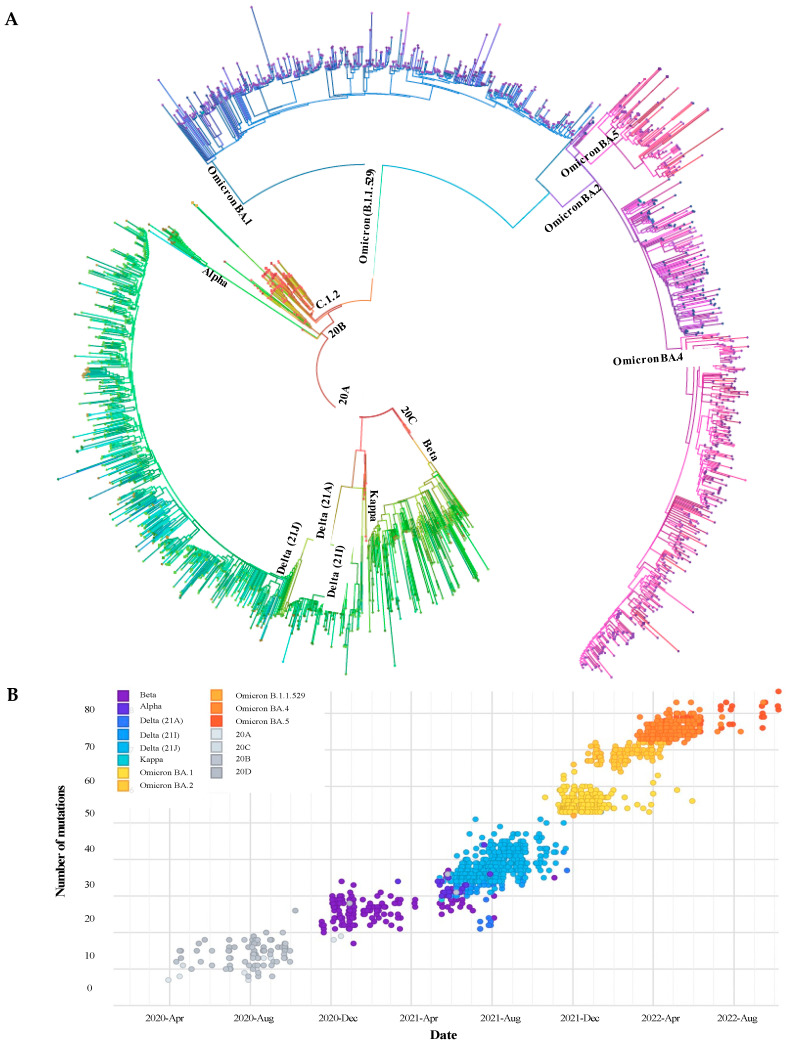
Phylogenetic analysis of SARS-CoV-2 lineages and VOCs detected in South Africa from 2020 to 2022. (**A**) Radial phylogenetic tree display of the lineages identified in South Africa. Tree rooted with the Wuhan-Hu1 strain. Our dataset was compared to the global database embedded in Nextstrain. (**B**) Nextclade custom time-tree displaying the increase in the number of mutations observed for each SARS-CoV-2 clade observed as time progressed.

Compared to global data, our sequences clustered with several VOCs, of which Beta, Delta and Omicron separated into the dominant waves of infection in South Africa as expected. All VOCs evolved from the clades 20C, 20A and 20B, respectively, after the first wave of infections (Figure 2A). Among our study samples, Alpha and Kappa were present, with 20B and 20A being the closest ancestors, respectively. An increase in the number of mutations gained across the SARS-CoV-2 whole genome was observed as new variants and their sub-lineages emerged from 2020 to 2022 (Figure 2A,B). From April to October 2020, 20A, 20B and 20C SARS-CoV-2 clades were dominant and characterised by ~8–30 mutations across their genomes. The number of mutations present in Beta increased from ~20 to 37 from November 2020 to June 2021, respectively. Several Delta sub-lineages were observed from June to November 2021, with Delta 21A having the lowest number of mutations (21–29) and Delta 21J with up to 51 mutations. Omicron B.1.1.529 had ~52–62 mutations; its sub-lineages BA.1 and BA.2 had up to 72 mutations, while BA.4 and BA.5 detected from March to October 2022 had 72 to >80 mutations.

### 3.4. Genome-Wide Diversity of Low-Frequency Lineages

**Figure 3:**
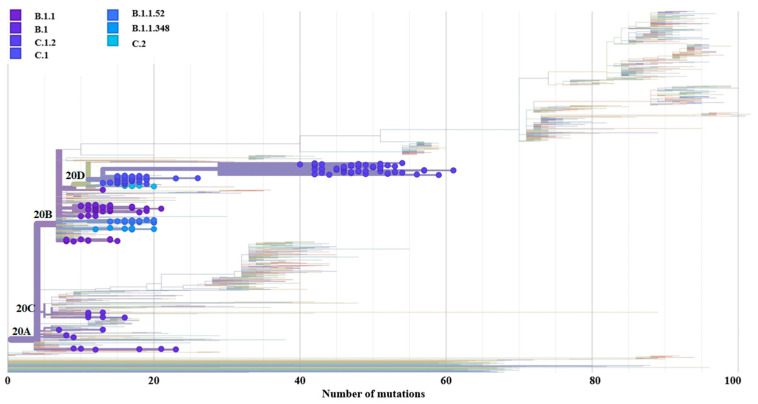
SARS-CoV-2 low-frequency lineages identified in this study. The blue-shaded circles represent the samples designated as low-frequency lineages from our study cohort. The horizontal clusters in the background represent the additional lineages and VOCs identified globally.

Seven percent (182/2547) of samples comprised low-frequency lineages, including B.1 (0.7%; 17/2547), B.1.1 (1.8%; 47/2547), B.1.1.348 (0.3%; 8/2547), B.1.1.52 (0.5%; 13/2547), C.1 (1.5%; 38/2547), C.1.2 (2.1%; 53/2547), and C.2 (0.2%; 6/2547). B.1 clustered with the 20A and 20C clades, B.1.1/B.1.152/B.1.1.348 clustered with the 20B clade, and C.1/C.1.2/C.2 clustered with the 20D clades (Figure 3). As new lineages emerged with time, an increase in the number of mutations was gained across the SARS-CoV-2 whole genome (Figure 3). The B.1 lineage was observed from 2020 through 2021. It was initially identified in March 2020 with 12 mutations which gradually increased to 21 mutations towards the end of April 2020.

**Figure 4:**
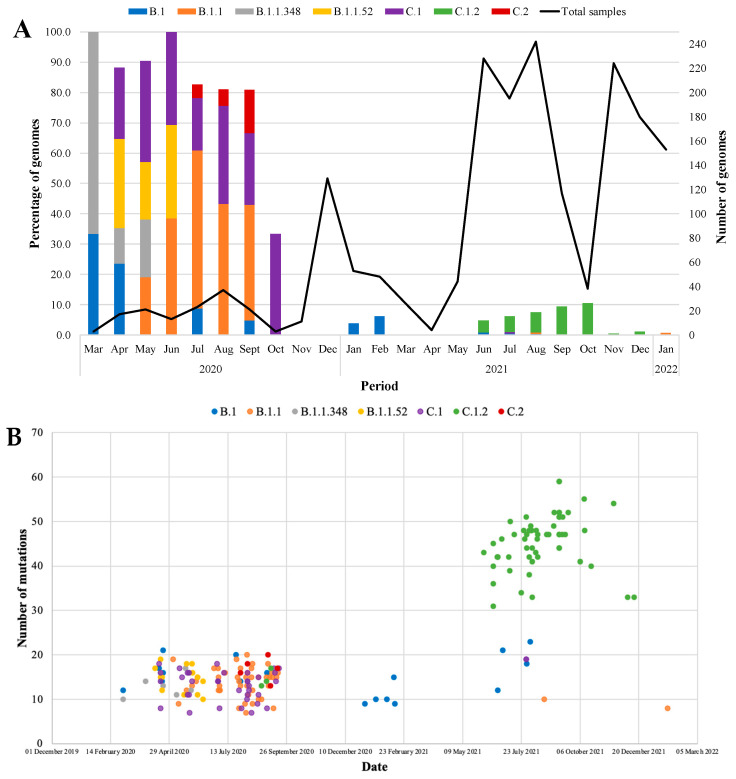
SARS-CoV-2 low-frequency lineages identified over time. (**A**) The bars represent the SAR-CoV-2 lineages, and the line graph represents the total number of samples that were successfully sequenced during each month from 2020 to 2022. (**B**) The shaded dots represent the samples designated as low-frequency lineages. The *x*-axis represents the total number of mutations identified in each lineage across the whole genome. The *y*-axis represents the date during which the lineages were observed.

B.1 was only observed again in July 2020, with 20 mutations and decreased to 16 mutations by September 2020 (Figure 4B, Table S5). In January and February 2021, B.1 only had 9–15 mutations and evolved further from June (21 mutations) to August (23 mutations). From 2020 to 2021, during the first three waves of infection, B.1 retained five mutations, including P323L and G671I in the RdRp protein; and C136F, delY144, and D614G in the S protein (Table S5). The B.1 lineage was predominantly observed among adults 45–60 years of age diagnosed through community screening and test services (3/7; 57.1%) and out-patients (2/7; 28.6%) (Table S1). B.1.1 was observed consistently from May to September 2020, with mutations fluctuating daily, with at least 7 mutations present and a maximum of 20 mutations (Figure 4B, Table S6). Six mutations carried over from 2020 to 2021 (during the 1st, 3rd and 4th waves), including R203K and G204R in the N protein; H208Y in the nsp2 protein; Y138H in the nsp8 protein; R126S in the ORF3a protein; P323L in the RdRp protein; and D614G in the S protein (Table S6). This lineage was predominant among younger adults aged 25–44 years from community screening (14/28; 50.05%) and out-patients in casualty (8/28; 28.6%) (Table S1).

B.1.1.348 was observed from March to May 2020, with <20 mutations seen throughout this period, with 10 mutations gained in March, 16 in April, and 17 in May (Figure 4B, Table S7). Six mutations were observed during the first wave, including S212R in the M protein, R203K and G204K in the N protein, del141-143 in the nsp1 protein, P323L in the RdRp protein, and D614G in the S protein. B.1.1.348 was predominantly detected in 25–44-year-old adults from community screening (Table S2). B.1.1.52 was present from April to June 2020, with a maximum of 19 mutations present in April 2020, which was stable until May 2020 (16–18 mutations) and declined by June 2020 (10–15 mutations) (Figure 4B, Table S8). Nine mutations were identified in B.1.1.52 during the first wave. Mutations included H210R and S212R in the M protein, R203K and G204R in the N protein, Y138H in the nsp8 protein, T1639A in the PLpro protein, P323L in the RdRp protein, and D614G in the S protein. More than 50% of individuals were infected with B.1.1.52 (7/13; 53.8%) among community screening and out-patients from ARV clinics and casualty (Table S2). D614G was the only S protein mutation that was present with more than a single occurrence among the B.1.1.348 (87.5%; 7/8) and B.1.1.52 (92.9%; 13/14) lineages. 

C.1 emerged in April 2020 and circulated until September 2020. The aforementioned lineage had <20 mutations overall, but >15 mutations were observed throughout the year and were present again in July 2021 with 19 mutations (Figure 4B, Table S9). During the 1st, 2nd and 3rd waves from 2020 to 2021, 7 mutations were retained in the C.1 lineage: G15S in the 3CLpro; R203K, G204R, and Q384H in the N protein; del255 in the ORF3a, T428I in the PLpro, and P323L in the S protein. C.1 was predominantly identified among young adults 25–44 years of age (22/38; 57.9%), primarily from community screening and out-patients from ARV clinics, healthcare workers and casualty (Table S3). C.1.2 was observed from June to December 2021 with 31 to 59 mutations, increasing rapidly from August to September 2021 (Figure 4B, Table S10). The C.1.2 samples had ~23 mutations in the S protein, of which >80% of sequences. In 2021, during the 3rd to 4th waves, several mutations carried over including the following: G15S and T24I in the 3CLpro, L21I and S68F/P in the E protein; L29F and I82T in the M protein; P13L, R203K, G204R and Q384H in the N protein; E102K in the nsp1 protein; L438P in the nsp4 protein; del106–108 in the nsp6 protein; S216L, T229N, del255 and L275F in the ORF3a; T428I and T819I in the PLpro protein, and P323L in the RdRp protein; P9L, P25L, C136F, delY144, R190S, D215G, del243-244, Y449H, E484K, N501Y, L585F, D614G, H655Y, N679K, T716I, and T859N in the S protein (Figure 4B, Table S10). In 2020, C.2 had 16 mutations present in July, which increased to 18 in August and 20 at the beginning of September and lost mutations in the middle of September (13–17 mutations) (Figure 4B). Lastly, C.2 only had two mutations, including D614G and N679K (Table S11). In 2020, during the first wave, eight mutations were detected in C.2. Mutations included G15S in the 3CLpro protein, R203K and G204R in the N protein, A85S in the nsp14 protein, T428I in the PLpro protein, L302S and P323L in the RdRp, and D614G in the S protein. Similar to other low-frequency lineages, C.1.2 and C.2 were predominant among adults aged 25–44 years from community surveillance (24/29; 82.8% and 1/3; 33.3%, respectively) and out-patients (4/29; 13.8% and 1/3; 33.3%, respectively).

## 4. Discussion

The SARS-CoV-2 global pandemic is fueled by several variants of concern or interest that displayed more efficient transmission or immune evasion properties. As VOCs evolved from the Wuhan-Hu1, SARS-CoV-2 has undergone convergence resulting in novel lineages that could have evolved from previous lineages or VOCs [1]. Our study describes the diversity of SARS-CoV-2 lineages and VOCs from the first to fifth COVID-19 waves in SA and the significance of lineages observed at low frequencies.

SARS-CoV-2 infected individuals were predominantly observed among younger adults aged 25–60 years and females, with no noticeable difference in the prevalence from 2020, 2021 or 2022. Globally, similar observations were made, with higher rates of infection observed among younger adults >25 years of age, with the majority of resultant deaths observed among older adults >60 years [18,19,20,21,22]. The detection rate of SARS-CoV-2 almost doubled for females (60.1%) when compared to males (37.5%). Of note was that the majority of cases were from the City of Johannesburg Metro district in Gauteng, which is expected since it is the hub of major public hospitals in Johannesburg, clinics and a variety of SARS-CoV-2 community screening sites. Approximately 72% of samples sequenced belonged to individuals that sought community screen and test services, followed by out-patients, and therefore history and symptom profiles of these individuals were not recorded as was for in-patients. Of note was that community screening was 30.7% and 20.6% greater in 2022 compared to 2020 and 2021, respectively.

We identified 24 lineages, apart from the dominating VOCs in SA, from 2020 to 2022, which descended from the Wuhan-Hu1 strain, including B.1 (3%; 8/278), B.1.1 (16%; 45/278), B.1.1.1 (2%; 5/278), B.1.1.348 (3%; 8/278), B.1.1.52 (5%; 13/278), C.1 (13%; 37/278), and C.2 (2%; 6/278), among which the D614G mutation was highly prevalent. Overall for SA, B1.1, B.1.1.52 and C.1 were detected at frequencies of 11%, 14% and 23%, respectively, before the emergence of Beta in 2020 [23,24], which is higher than observed for Gauteng in this study except for B.1.1. These three lineages (B.1, B.1.1 and C.1) were the only low-frequency lineages that were not completely displaced and circulated during more than one wave. The B.1 lineage was predominant across Africa at the beginning of the pandemic [3]. B.1 gained up to 21 mutations across the genome over time from 2020 to 2021. B.1 was detected at a high prevalence of 46% in the United States of America (USA) and 11% in Turkey, whereas for Africa, it was 26.7% compared to 2% overall for SA and 3% in this study [25,26]. This lineage was responsible for the 2020 SARS-CoV-2 outbreak in Northern Italy [27]. Similar to our study, B.1 was detected in Spain through waves 2 to 4 [28]. B.1 was detected in less than 1% of SARS-CoV-2 genomes from Peru in 2021, whereas it was detected at a high frequency of 46.3% in Colombia [29,30].

The number of mutations in B.1.1 decreased from 20 to 8 by 2022. This lineage dominated in the UK at 25% and at 17% in the USA [25,26]. B.1.1 was also reported as one of the dominant (32.9%) SARS-CoV-2 lineages in Spain during the first wave in 2020, whereas B.1 was present at 2.7% during the same time [28]. In Peru, B.1.1 was detected at a frequency of 1%, while in Chile, it was reported as one of five lineages that dominated early in the pandemic [30,31]. B.1.1.348 was detected in Argentina during the first wave of SARS-CoV-2 infections at a low frequency of 3.7% and was present in Peru at 3% and at a higher frequency of 9.7% in Colombia [30,32,33]. Chile also reported B.1.1.348 as one of five dominant lineages early in the pandemic [31]. B.1.1.348 was also detected in Spain in a few cases [28].

Globally C.1, C.1.2 and C.2 had a cumulative prevalence of <0.5%, with SA accounting for 94.6% (437/462), 89.5% (307/343), and 45.8% (33/72) of the data, respectively [25,26]. C.1 had ~16 mutations in 2020 which remained somewhat consistent with up to 19 mutations by 2021, which was similar to findings in previous studies from SA with the majority of sequences geographically representative of KwaZulu-Natal and the Free State Provinces [10,34]. However, the latter study detected B.1.1.54 (28.8%; 320/1 111) and B.1.1.56 (9.4%; 104/1 111) lineages (~14 mutations) from the parent lineage B.1.1 at high prevalence during the first wave across five SA provinces, but the majority of sequences were from KwaZulu-Natal [10]. Whereas the B.1.1.53, B.1.1.54, B.1.1.117, B.1.237 and B.1.381, from the parent lineage B.1 and B.1.1, were the most prevalent in the Free State but were not detected in our study [34]. This may suggest that B.1.1.348, B.1.1.52 and C.2 were more specific to Gauteng, especially within the City of Johannesburg Metro. C.1.2 (20D clade) evolved from the C.1 lineage [11] and circulated at low frequencies in 2021 during the Delta wave. C.1.2 was a variant of interest for SA during 2021. It was only observed from June to December 2021 at a frequency of <10% during each month but never outcompeted other VOCs. C.1.2 was observed across all nine provinces of SA with <4% monthly prevalence from November 2021. While our study showed a slightly higher prevalence of 6.1% (53/870) and represented ~32% of all the South African C.1.2 strains described [11], C.1.2 had 23 highly prevalent mutations that occurred concurrently (co-evolved), with the exception of L585F (17%) and D936H (5%) observed in a previous study [11]. Significant mutations in the S protein were detected in lower frequency lineages and also characteristic in VOCs that were dominant in SA. Mutations including D614G, D215G, N440K, S477N, T478K, E484K and H655Y are responsible for enhancing ACE2 binding affinity, transmissibility or immune escape [15,35]. The low-frequency lineages were primarily observed among young adults aged 25–44 years of age from community screening and out-patients. Since a very limited number of in-patients were identified with the B and C lineages, this suggests that they were not associated with severe illness.

There were several important mutations worth noting that were detected during more than one SARS-CoV-2 wave among the low-frequency lineages in this study. The S68F substitution in the E protein can also assist with stabilising the protein structure [36,37]. A significant M protein mutation was the I82T, which is involved in increasing structural stability and was most prevalent in the B.1.575 lineages in the USA in 2020 [38,39]. The N protein mutations, including P13L, R203K and G204R/K, may assist with increasing the transmission of the SARS-CoV-2 but also be associated with reduced severity of disease and, therefore, lower mortality rates when compared to individuals with the wild-type N protein [40,41,42]. In the nsp1 protein, the E102K mutation encourages attachment to viral RNA, increasing replication and thereby increasing infectivity [43]. While del141-143 in nsp1, also reported in Omicron BA.4 lineages during the fifth wave in SA and described in Delta AY.63 in Norway, extends the duration of infection [5,44]. R126S in ORF3a compromises the viral structure by reducing its stability [45]. P323L was observed across all low-frequency lineages and was previously described as responsible for maintaining the RdRp protein structure and may enhance mutation rates [46,47]. Among the S protein mutation in the low-frequency lineages, the most significant were the following: N501Y which encourages affinity to human ACE-2 [48]; del69-70 and delY144 may influence the antigenic properties protein (NTD); furthermore, del69-70 escapes neutralising antibodies, increases viral replication and transmissibility [48,49,50,51]; E484K assists the virus by escaping host immunity and increased binding affinity to the hosts ACE-2 receptor [51]; D614G was identified in all VOCs and found to improve ACE-2 receptor binding thereby increasing viral transmissibility [52,53,54]; H655Y was also detected in Gamma and Omicron, and increases virulence by encouraging furin cleavage, evading immunity and increasing transmission; N679K increases glycosylation at the S1/S2 furin cleavage site which could prevent syncytia formation [40,55].

The circulation of SARS-CoV-2 VOC were similar to that reported previously in SA during the first five COVID-19 waves [56,57]. Wuhan-Hu1 ancestral strains driving the first wave (2020: epiweeks 14–42), Beta (2020: epiweeks 43 to 2021: epiweek 25), Delta (2021: epiweeks 20 to 45), Omicron BA.1/BA.2 (2021: epiweek 46 to 2022: epiweek 12) and Omicron BA.4/BA.5 (2022: epiweeks 13 to 42) driving the 2nd, 3rd, 4th and 5th waves, respectively. Several studies have previously reported on the circulation of VOCs in SA, which, except for Alpha, drove the major waves of infection from 2020 to 2022 [2,3,15,58,59]. We identified Alpha in 2021 between the Beta and Delta waves; however, it was never a dominant lineage and only circulated scantly across 9 weeks. Beta evolved from the B.1.1 lineage (20C clade), with up to 37 mutations gained across the genome by the end of the second wave. After its initial detection in SA, Beta spread across 20 countries in Africa [3]. This VOC had eight mutations in the S protein, with the most significant being K417N, E484K and N501Y within the RBD [2,25,60]. Beta was not completely displaced after the second wave since we detected it at low frequencies later in 2021 towards the tail-end of the Delta wave, as well as in several other provinces in SA and <0.1% globally [56,61].

We identified 14 different Delta sub-lineages, with AY.45 dominating from epiweeks 20 to 45 in 2021. Similarly, Delta AY.45 (21J clade) had an overall prevalence of 80% (522/650) by the end of October 2021 (epiweek 45), with Western Cape being the only province with a similar prevalence of B.1.617.2 (21A clade) and AY.45 [56]. Phylogenetic analysis showed that Delta descended from the B.1 lineage (20A clade) and gained up to 51 mutations across the genome by the end of the third wave, with 9–10 mutations in the S protein [60]. L452R and P681R were the common mutations identified in Delta sub-lineages, while T478K was the primary mutation in the S protein among the Delta lineages, which is responsible for interacting with the human ACE-2 receptor, increasing its infectivity [25,60,62]. From our study, Beta and Delta circulated for up to 30 consecutive weeks; however, the detection rate of Delta was much greater than Beta, suggesting Delta has higher rates of viral replication and transmission [62].

Omicron outcompeted Delta unexpectedly and at rapid rates in 2021, with a detection rate of 30.2% (422/1399) in 2021 and 100% (870/870) in 2022. Similar events were observed globally [25]. Phylogenetic analysis showed that Omicron lineages and sub-lineages disseminated from the 20B clade. We identified 37 Omicron sub-lineages from epiweek 43 in 2021 to epiweek 42 in 2022, including several recombinants (BF.28, CP.5, XAB, XAM, XAR, XAS, XAY and XT) sporadically detected with a prevalence of 2.4% (21/870). BQ.1, XD, XE, XF and XBB recombinants also identified in our study were reported to display a significant increase in infectivity and immune evasion [63,64]. As Omicron evolved from BA.1 to BA.5, the virus gained >80 mutations across its genome, up to 34 mutations in the S protein. Evidence of convergence was present in Omicron, which carried over a few important mutations from Beta (K417N, N501Y), Alpha (P681R and del69/70) and Delta (T478K and P681H) [60,65,66]. These important mutations were known to increase the spike trimer stability and were present in the absence of host immune pressure [66]. BA.1 was outcompeted by BA.2, and the del69/70 fell away, which was the distinguishing feature between BA.1 and BA.2 [5,67]. Omicron BA.2 was then outcompeted by BA.4 and BA.5, which gained the del69/70, L452R, F486V and Q493 wild type [5].

The main limitation identified in this study was the inconsistency of sequence data in 2020. The number of genomes sequenced in 2020 was limited since routine surveillance commenced late in 2020, and retrospective sequencing had to be performed. However, our dataset proves to be a good representation due to random selection, as the major VOC events observed in SA are reported accurately when compared to the national surveillance data. In addition, the emergence of novel lineages and VOC primer mismatches resulted in challenges in obtaining high-quality sequence data with adequate coverage across the genome. To overcome this, primer optimisation was required to prevent incorrect lineage/VOC assignment. Therefore, in this study, only genome sequences with 65–99% coverage across the genome and with >80% coverage for the S proteins were included.

## 5. Conclusions

Our study showed that with the emergence of novel lineages and VOCs, the number of mutations increased simultaneously, reducing protection against vaccine-induced or natural immune response and other environmental selective pressures. In addition, we identified that B.1, B.1.1 and C.1 lineages were able to retain mutations over time and still maintained their standing during the five COVID-19 waves in SA, although at much lower frequencies. These lineages presented with mutations that were carried over with the VOCs. It is possible that low-frequency lineages, together with VOCs circulating, can lead to possible convergence and recombination, which may result in the next novel lineage or variant that may further increase transmissibility, infectivity and escape vaccine-induced or natural host immunity. Therefore, it is important that low-frequency variants be studied in conjunction with VOCs across the globe to determine their impact on different populations.

## Figures and Tables

**Table 1:** Demographic characteristics of successfully sequenced SARS-CoV-2 samples from 30 March 2020 to 13 October 2022 (*n* = 2547).

Demographics	Years			Total (*n* = 2547)
	2020 (*n* = 278)	2021 (*n* = 1399)	2022 (*n* = 870)	
**Age Groups (*n* (%))**				
<5	4 (1.4%)	12 (0.9%)	15 (1.7%)	31 (1.2%)
5–14	6 (2.2%)	129 (9.2%)	54 (6.2%)	189 (7.7.%)
15–24	26 (9.4%)	234 (16.7%)	119 (13.7%)	379 (15.4%)
25–44	141 (50.7%)	611 (43.7%)	410 (47.1%)	1162 (47.3%)
45–60	59 (21.2%)	296 (21.2%)	162 (18.6%)	517 (21.0%)
>60	33 (3.2%)	97 (6.9%)	68 (7.8%)	198 (8.1%)
Unknown *	9 (3.2%)	20 (1.4%)	42 (4.8%)	71 (2.9%)
**Sex (*n* (%))**				
Female	169 (60.8%)	813 (58.1%)	548 (63.0%)	1531 (60.1%)
Male	106 (38.1%)	550 (39.3%)	299 (34.4%)	956 (37.5%)
Unknown *	3 (1.1%)	36 (2.6%)	23 (2.6%)	62 (2.4%)
**Province (*n* (%))**				
Eastern Cape	5 (1.8%)	54 (3.7%)	0 (0.0%)	59 (2.3.%)
Gauteng	264 (95.0%)	1337 (95.6%)	870 (100.0%)	2471 (97.0%)
KwaZulu-Natal	7 (2.5%)	1 (0.1%)	0 (0.0%)	8 (0.3%)
Limpopo	0 (0.0%)	1 (0.1%)	0 (0.0%)	1 (0.1%)
Western Cape	0 (0.0%)	6 (0.4%)	0 (0.0%)	6 (0.4%)
Unknown *	2 (0.7%)	0 (0.0%)	0 (0.0%)	2 (0.1%)
**Patient Status (*n* (%))**				
Community screening and test	142 (51.5%)	1150 (82.2%)	536 (61.6%)	1828 (71.8%)
Deceased	0 (0.0%)	4 (0.3%)	11 (1.3%)	15 (0.6%)
In-patient	40 (14.4.%)	77 (5.5%)	92 (10.6%)	209 (8.2%)
Out-patient	93 (33.5%)	168 (12.0%)	231 (26.6%)	492 (19.3%)
Unknown *	3/278 (1.1%)	0 (0.0%)	0 (0.0%)	3/2459 (0.1%)

* Unknown represents those individuals for which the data was unavailable or not recorded at the time of specimen collection.

## Data Availability

All genome sequences and associated metadata in this dataset are published in GISAID’s EpiCoV database. GISAID Identifier EPI_SET_230215tf (https://epicov.org/epi3/epi_set/230215tf?main=true) included 2547 sequences included in this study. The GISAID Identifier EPI_SET_221129ph (https://epicov.org/epi3/epi_set/221129ph?main=true) for 182 individual genome sequences that comprised the low-frequency lineages located.

## Supplementary Materials

The following supporting information can be downloaded at: https://www.mdpi.com/1999-4915/15/5/1194/s1, Table S1: Demographic data for B.1 and B.1.1 lineages from 2020 to 2022; Table S2: Demographic data for B.1.1.438 and B.1.1.52 lineages from 2020 to 2022; Table S3: Demographic data for C.1 and C.1.2 lineages from 2020 to 2022; Table S4: Demographic data for C.2 lineages from 2020 to 2022; Table S5: Mutations detected across the whole genome of B.1 lineage from 2020 to 2021; Table S6: Mutations detected across the whole genome of B.1.1 lineage from 2020 to 2022; Table S7: Mutations detected across the whole genome of B.1.1.348 lineage in 2020; Table S8: Mutations detected across the whole genome of B.1.1.52 lineage from 2020; Table S9: Mutations detected across the whole genome of C.1 lineage from 2020 to 2021; Table S10: Mutations detected across the whole genome of C.1.2 lineage from 2020 to 2021; Table S11: Mutations detected across the whole genome of C.2 lineage from 2020 to 2021.
