# Correction: Subramoney et al. Molecular Epidemiology of SARS-CoV-2 during Five COVID-19 Waves and the Significance of Low-Frequency Lineages. *Viruses* 2023, *15*, 1194

**DOI:** 10.3390/v15071502

**Published:** 2023-07-04

**Authors:** Kathleen Subramoney, Nkhensani Mtileni, Jennifer Giandhari, Yeshnee Naidoo, Yajna Ramphal, Sureshnee Pillay, Upasana Ramphal, Akhil Maharaj, Derek Tshiabuila, Houriiyah Tegally, Eduan Wilkinson, Tulio de Oliveira, Burtram C. Fielding, Florette K. Treurnicht

**Affiliations:** 1 School of Pathology, Faculty of Health Sciences, University of the Witwatersrand, Johannesburg 2193, South Africa; florette.treurnicht@nhls.ac.za; 2 Department of Virology, National Health Laboratory Service, Charlotte Maxeke Johannesburg Academic Hospital, Johannesburg 2193, South Africa; 3 Centre for Vaccines and Immunology, National Institute for Communicable Diseases, Johannesburg 2131, South Africa; 4 KwaZulu-Natal Research Innovation and Sequencing Platform (KRISP), Nelson R Mandela School of Medicine, University of KwaZulu-Natal, Durban 4001, South Africa; 5 Centre for Epidemic Response and Innovation (CERI), School of Data Science and Computational Thinking, Stellenbosch University, Stellenbosch 7600, South Africa; 6 Molecular Biology and Virology Research Laboratory, Department of Medical BioSciences, University of the Western Cape, Cape Town 7535, South Africa

In the original publication [1], there was a mistake in Figure 1 as published. An error occurred occasionally, resulting in redundant horizontal lines in the bar graphs of 1A and 1C and the disappearance of the light yellow bars in 1C. The corrected Figure 1 appears below. The authors state that the scientific conclusions are unaffected. This correction was approved by the Academic Editor. The original publication has also been updated.

## Figures and Tables

**Figure 1:**
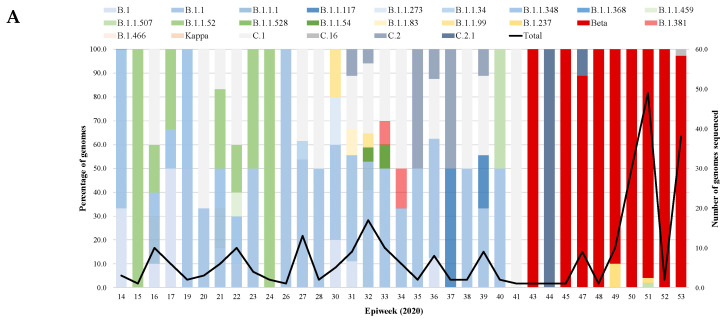

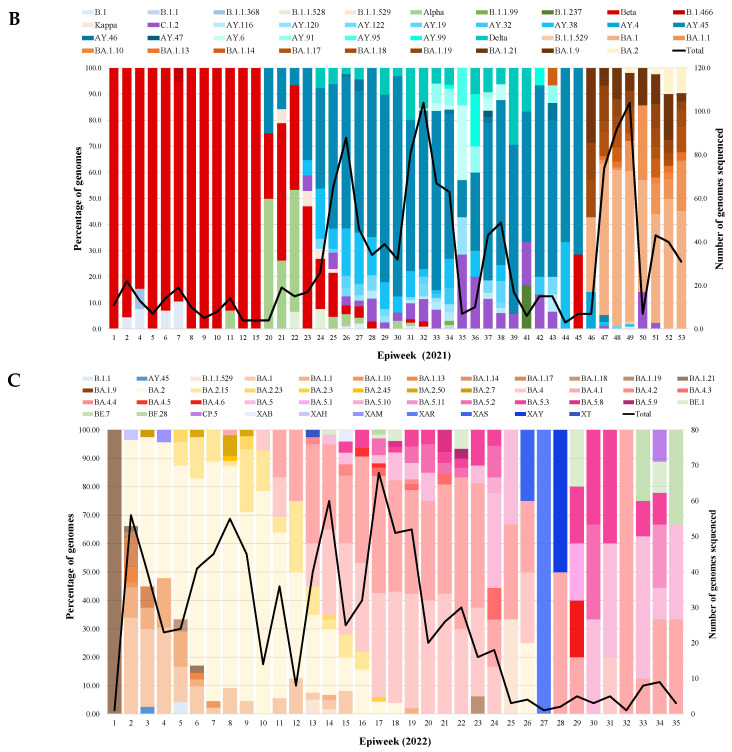
Prevalence of SARS-CoV-2 lineages over time from 2020 to 2022 by epiweek. The bar graph represents the SAR-CoV-2 lineages and VOCs identified in our study cohort. The black line graph represents the total number of samples that were sequenced during each epiweek. (**A**) Distribution of SARS-CoV-2 lineages in 2020, (**B**) Distribution of SARS-CoV-2 lineages in 2021, and (**C**) Distribution of SARS-CoV-2 lineages in 2022.
