# Supplementary material for: Molecular Epidemiology of SARS-CoV-2 during Five COVID-19 Waves and the Significance of Low-Frequency Lineages
Source: Viruses. 2023 May 18;15(5):1194. doi: 10.3390/v15051194 (PMC10223853; doi:10.3390/v15051194)
Supplement: Supplementary file 1 [file viruses-15-01194-s001.zip › Supplementary tables S1 to S4.pdf]

Supplementary data

Table S1. Demographic data for B.1 and B.1.1 lineages from 2020 to 2022

Patient status	B.1 (N=17)							B.1.1 (N=47)						
	<5 (N=0)	5-14 (N=1)	15-24 (N=0)	25-44 (N=5)	45-60 (N=7)	>60 (N=3)	Unknown (N=1)	<5 (N=0)	5-14 (N=0)	15-24 (N=6)	25-44 (N=28)	45-60 (N=7)	>60 (N=3)	Unknown (N=1)
**Community screen and test**		**1/1 (100%)**		**5/5 (100%)**	**3/7 (57.1%)**	**2/3 (66.7%)**				**2/6 (33.3%)**	**14/28 (50.0%)**	**5/9 (55.6%)**	**2/3 (66.7%)**	
**In-patient**					**2/7 (28.6%)**					**2/6 (33.3%)**	**2/28 (7.1%)**	**1/9 (11.1%)**		
Cardiac unit										1/6 (16.7%)	1/28 (3.6%)			
Neurology					1/7 (14.3%)									
Other					1/7 (14.3%)					1/6 (16.7%)	1/28 (3.6%)	1/9 (11.1%)		
**Out-patient**					**1/7 (14.3%)**					**1/6 (16.7%)**	**12/28 (42.9%)**	**3/9 (33.3%)**	**1/3 (33.3%)**	**1/1 (100%)**
ARV clinic											1/28 (3.6%)	1/9 (11.1%)		
Healthcare worker										1/6 (16.7%)	2/28 (7.1%)		1/3 (33.3%)	
Paediatric											1/28 (3.6%)			
Casualty					1/7 (14.3%)						8/28 (28.6%)	2/9 (22.2%)		
**Unknown**						**1/3 (33.3%)**	**1/1 (100%)**							
**Total**	**0/17 (0.0%)**	**1/17 (5.9%)**	**0/17 (0.0%)**	**5/17 (29.4%)**	**7/17 (41.2%)**	**3/17 (6.4%)**	**0/17 (0.0%)**	**0/47 (0.0%)**	**0/47 (0.0%)**	**6/47 (12.8%)**	**28/47 (59.6%)**	**9/47 (19.2%)**	**3/47 (6.4%)**	**1/47 (2.1%)**

Table S2. Demographic data for B.1.1.438 and B.1.1.52 lineages from 2020 to 2022

Patient status	B.1.1.348 (N=8)							B.1.1.52 (N=13)						
	<5 (N=0)	5-14 (N=0)	15-24 (N=1)	25-44 (N=3)	45-60 (N=2)	>60 (N=1)	Unknown (N=1)	<5 (N=1)	5-14 (N=1)	15-24 (N=0)	25-44 (N=7)	45-60 (N=3)	>60 (N=0)	Unknown (N=1)
**Community screen and test**			**1/1 (100%)**	**3/3 (100%)**	**1/2 (50.0%)**		**1/1 (100%)**	**1/1 (100%)**	**1/1 (100%)**		**2/7 (28.6%)**	**2/3 (66.7%)**		
**In-patient**											**1/7 (14.3%)**			
ICU											1/7 (14.3%)			
**Out-patient**					**1/2 (50.0%)**						**4/7 (57.1%)**	**1/3 (33.3%)**		**1/1 (100%)**
ARV clinic						1/1 (100%)					2/7 (28.6%)			
Healthcare worker					1/2 (50.0%)	1/1 (100%)						1/3 (33.3%)		1/1 (100%)
Casualty											2/7 (28.6%)			
**Total**	**0/8 (0.0%)**	**0/8 (0.0%)**	**1/8 (12.5%)**	**3/8 (37.5%)**	**2/8 (25.0%)**	**1/8 (12.5%)**	**1/8 (12.5%)**	**1/13 (7.7%)**	**1/13 (7.7%)**	**0/13 (0.0%)**	**7/13 (53.8%)**	**3/13 (23.1%)**	**0/13 (0.0%)**	**1/13 (7.7%)**

Table S3. Demographic data for C.1 and C.1.2 lineages from 2020 to 2022

Patient status	C.1 (N=38)							C.1.2 (N=53)						
	<5 (N=0)	5-14 (N=1)	15-24 (N=3)	25-44 (N=22)	45-60 (N=7)	>60 (N=3)	Unknown (N=1)	<5 (N=0)	5-14 (N=1)	15-24 (N=8)	25-44 (N=29)	45-60 (N=10)	>60 (N=5)	Unknown (N=0)
**Community screen and test**		**1/1 (100%)**	**3/3 (100%)**	**11/22 (50.0%)**	**1/7 (14.2%)**					**8/8 (100%)**	**24/29 (82.8%)**	**10/10 (100%)**	**4/5 (80.0%)**	
**In-patient**				**2/22 (9.1%)**	**2/7 (28.6%)**	**1/3 (33.3%)**			**1/1 (100%)**		**1/29 (3.5%)**			
Chronic dialysis					1/7 (14.3%)									
Other				2/22 (9.1%)	1/7 (14.3%)	1/3 (33.3%)			1/1 (100%)		1/29 (3.5%)			
**Out-patient**				**9/22 (40.9%)**	**4/7 (57.1%)**	**2/3 (66.7%)**					**4/29 (13.8%)**		**1/5 (20.0%)**	
ARV clinic				2/22 (9.1%)										
Healthcare worker				2/22 (9.1%)							1/29 (3.5%)			
Casualty				5/22 (22.7%)	4/7 (57.1%)	2/3 (66.7%)					3/29 (10.3%)		1/5 (20.0%)	
**Unknown**							**1/1 (100%)**							
**Total**	**0/38 (0.0%)**	**1/38 (2.6%)**	**3/38 (7.9%)**	**22/38 (57.9%)**	**7/38 (18.4%)**	**3/38 (7.9%)**	**1/38 (2.6%)**	**0/6 (0.0%)**	**1/6 (16.7%)**	**8/53 (15.1%)**	**29/53 (54.7%)**	**10/53 (18.9%)**	**5/53 (9.4%)**	**0/53 (0.0%)**

Table S4. Demographic data for C.2 lineages from 2020 to 2022

**Patient status**	**C.2 (N=6)**						
	**<5 (N=0)**	**15-24 (N=1)**	**15-24 (N=0)**	**25-44 (N=3)**	**45-60 (N=1)**	**>60 (N=1)**	**Unknown (N=0)**
**Community screen and test**		**1/1 (100%)**		**1/3 (33.3%)**			
**In-patient**				**1/3 (33.3%)**	**1/1 (100%)**		
Oncology					**1/1 (100%)**		
Rheumatology/nephrology				**1/3 (33.3%)**			
**Out-patient**				**1/3 (33.3%)**		**1/1 (100%)**	
Casualty				1/3 (33.3%)		1/1 (100%)	
**Total**	**0/6 (0.0%)**	**1/6 (16.7%)**	**0/6 (0.0%)**	**3/6 (50.0%)**	**1/6 (16.7%)**	**1/6 (16.7%)**	**0/6 (0.0%)**
