# Supplementary material for: Molecular Epidemiology of SARS-CoV-2 during Five COVID-19 Waves and the Significance of Low-Frequency Lineages
Source: Viruses. 2023 May 18;15(5):1194. doi: 10.3390/v15051194 (PMC10223853; doi:10.3390/v15051194)
Supplement: Supplementary file 1 [file viruses-15-01194-s001.zip › Supplementary tables S5 to S11.pdf]

Table S5. Mutations detected across the whole genome of B.1 lineage from 2020 to 2021

Genes	3CLpro	E	M					N					nsp2			nsp4			
**Positions**	90	71	3	23	82	210	212	63	194	203	205	377	267	339	596	37	167	399	492
**Wuhan Hu-1**	K	P	D	V	I	H	S	D	S	R	T	D	N	G	A	T	V	K	T
March 2020 (N=11)																			
April 2020 (N=4)						R	R									S			
July 2020 (N=2)			G						L				del						
September 2020 (N=1)				L					L										
January 2021 (N=2)	R	L																	
February 2021 (N=2)	R			L							I			S					
June 2021 (N=2)					T							Y					L		
July 2021 (N=1)					T			G		M		Y							
August 2021 (N=1)					T					M		Y			T				I

Genes	nsp10	nsp13	nsp14	ORF3a		ORF7a		ORF7b	ORF8		PLpro							RdRp					
**Positions**	48	77	316	26	57	82	120	40	119	120	250	428	488	1381	1382	1639	1887	323	554	555	556	557	671
**Wuhan Hu-1**	H	P	V	S	Q	V	T	T	D	F	G	T	A	A	K	T	K	P	A	R	T	V	G
March 2020 (N=11)																A	N	L					
April 2020 (N=4)	Y				H													L					
July 2020 (N=2)																		L					
September 2020 (N=1)			F															L					S
January 2021 (N=2)																		L	V				
February 2021 (N=2)					H											A		L					
June 2021 (N=2)		L					I		del	del		I						L					
July 2021 (N=1)		L				A		I	del	del	R							L					
August 2021 (N=1)		L		L					del	del			S	D	S			L					S

Genes	S																						
**Positions**	80	98	136	144	156	157	158	190	215	243	244	417	449	452	478	484	501	614	679	701	716	859	950
**Wuhan Hu-1**	D	S	C	Y	E	F	R	R	D	A	L	K	Y	L	T	E	N	D	N	A	T	T	D
March 2020 (N=11)																		G					
April 2020 (N=4)		F																G					
July 2020 (N=2)		F																G					
September 2020 (N=1)			F	del				S	G									G					
January 2021 (N=2)	A																	G		V			
February 2021 (N=2)	A																	G		V			
June 2021 (N=2)	A		F	del				S	G	del	del		H			K	Y	G	K		I	N	
July 2021 (N=1)			F	del											K			G					
August 2021 (N=1)	A				del	del	G							R				G					N

*3C-like protease (3CLpro), envelope (E), nucleocapsid (N), non-structural protein (nsp), open-reading frame (ORF), papain-like protease (PLpro), RNA-dependent RNA-polymerase RdRp, spike (S)

Table S6. Mutations detected across the whole genome of B.1.1 lineage from 2020 to 2022

Genes	3CLpro			E			N							nsp2						nsp4				nsp6				nsp8	nsp13	nsp14				nsp15		nsp16
**Positions**	15	89	142	58	19	212	141	145	179	199	203	204	371	57	208	385	537	550	624	83	218	446	457	37	58	168	188	138	85	138	177	324	349	54	257	160
**Wuhan Hu-1**	G	L	N	V	Q	S	T	H	G	P	R	G	D	E	H	A	Y	L	P	T	S	A	A	L	M	I	G	Y	A	A	L	D	K	A	R	K
May 2020 (N=1)	S		S			R					K	R					D				F							H								
June 2020 (N=1)		F	S								K	R								I								H					N	V		
July 2020 (N=1)									V		K	R	Y		Y				L					F				H		V		Y				
August 2020 (N=1)				I	E						K	R		G		V									I			H	V				N		C	R
September 2020 (N=1)							I	N			K	R			Y			F				S	V			T		H	V		F					
August 2021 (N=1)											K	R			Y												S	H								
January 2022 (N=1)										S	K	R																H								

Genes	ORF3a							ORF7b											ORF8		Plpro								RdRp			S																				
**Positions**	15	23	54	121	126	172	191	4	5	6	7	8	9	10	11	12	13	14	11	28	126	236	341	493	503	1119	1285	1639	173	257	323	12	69	70	78	98	142	156	157	158	339	417	440	477	478	484	549	554	575	614	655	698
**Wuhan Hu-1**	L	A	A	V	R	G	E	L	S	L	I	D	F	Y	L	C	F	L	T	A	S	P	I	E	P	F	S	T	R	V	P	S	H	V	R	S	G	E	F	R	G	K	N	S	T	E	T	E	A	D	H	S
May 2020 (N=1)							A																			I		A		F	L																			G		
June 2020 (N=1)					S																						Y			F	L																			G		
July 2020 (N=1)					S		A																	A							L				M	F												D		G		
August 2020 (N=1)		S			S		A												K			S	V		S						L		del	del			D	del	del	G		N	K	N	J	A	I			G	Y	L
September 2020 (N=1)	F		S	L	S	C		del	del	del	del	del	del	del	del	del	del	del	K		L			A					C		L	F											K						S	G		
August 2021 (N=1)																															L																			G		
January 2022 (N=1)																				V											L										D									G		

*3C-like protease (3CLpro), envelope (E), nucleocapsid (N), non-structural protein (nsp), open-reading frame (ORF), papain-like protease (PLpro), RNA-dependent RNA-polymerase RdRp, spike (S)

Table S7. Mutations detected across the whole genome of B.1.1.348 lineage in 2020

Genes	E	M			N		nsp1			nsp2	nsp4	PLpro		RdRp	S	
**Positions**	19	125	210	212	203	204	141	142	143	424	492	428	1639	323	95	614
**Wuhan Hu-1**	L	H	H	S	R	G	K	S	F	G	T	T	T	P	T	D
March 2020 (N=2)				R	K	R	del	del	del	C				L		G
April 2020 (N=2)		P	R	R	K	R						I	A	L		G
May 2020 (N=4)	P			R	K	R	del	del	del	C	A		A	L	I	G

*envelope (E), membrane (M), nucleocapsid (N), non-structural protein (nsp), papain-like protease (PLpro), RNA-dependent RNA-polymerase RdRp, spike (S)

Table S8. Mutations detected across the whole genome of B.1.1.52 lineage from 2020

Genes	3CLpro		M		N		nsp1	nsp2	nsp5	nsp6	nsp8	nsp14	orf3a		Plpro		RdRp		S
**Positions**	**142**	**196**	**210**	**212**	**203**	**204**	**84**	**424**	**218**	**220**	**138**	**349**	**23**	**255**	**666**	**1639**	**257**	**323**	**614**
**Wuhan Hu-1**	**N**	**T**	**H**	**S**	**R**	**G**	**V**	**G**	**S**	**F**	**Y**	**K**	**A**	**V**	**T**	**T**	**V**	**P**	**D**
April 2020 (N=5)		M	R	R	K	R		C	F		H		S	del	S	A		L	G
May 2020 (N=4)	S		R	R	K	R		C		V	H					A	F	L	G
June 2020 (N=4)			R	R	K	R	I				H	N		del		A		L	G

*3C-like protease (3CLpro), membrane (M), nucleocapsid (N), non-structural protein (nsp), open-reading frame (ORF), papain-like protease (PLpro), RNA-dependent RNA-polymerase RdRp, spike (S)

Table S9. Mutations detected across the whole genome of C.1 lineage from 2020 to 2021

Genes	3CLpro		M		N								nsp1		nsp2	nsp4			nsp6	nsp8			nsp13			nsp14		ORF3a				ORF8		PLpro			RdRp	S
**Positions**	**15**	**38**	**8**	**68**	**34**	**70**	**141**	**203**	**204**	**272**	**382**	**384**	**49**	**156**	**200**	**218**	**438**	**457**	**37**	**29**	**138**	**178**	**18**	**392**	**403**	**305**	**516**	**202**	**240**	**254**	**255**	**24**	**62**	**428**	**945**	**1639**	**323**	**98**
**Wuhan Hu1**	**G**	**C**	**I**	**A**	**G**	**Q**	**T**	**R**	**G**	**Q**	**L**	**Q**	**G**	**D**	**P**	**S**	**L**	**A**	**L**	**G**	**Y**	**P**	**A**	**R**	**A**	**I**	**T**	**V**	**P**	**G**	**V**	**S**	**V**	**T**	**K**	**T**	**P**	**S**
April 2020 (N=4)	S			del				K	R		S	H		N		F					H										del			I		A	L	
May 2020 (N=7)	S	G					I	K	R			H	C	N	S																del			I		A	L	
June 2020 (N=4)	S		V					K	R	R		H												C							del			I			L	
July 2020 (N=4)	S				W			K	R			H							F			S			V	V					del		M	I			L	
August 2020 (N=12)	S					R		K	R			H					F	T	F	S			T				I			R	del			I	R		L	
September 2020 (N=5)	S					R		K	R			H																del	S		del			I	R		L	F
October 2020 (N=1)								K	R																						del			I		A	L	
July 2021 (N=1)	S							K	R			H																			del			I			L	

*3C-like protease (3CLpro), membrane (M), nucleocapsid (N), non-structural protein (nsp), open-reading frame (ORF), papain-like protease (PLpro), RNA-dependent RNA-polymerase RdRp, spike (S)

Table S10. Mutations detected across the whole genome of C.1.2 lineage from 2020 to 2021

Genes	3CLpro				E		M		N							nsp1							
**Positions**	**15**	**24**	**90**	**108**	**21**	**68**	**29**	**82**	**13**	**80**	**186**	**203**	**204**	**383**	**384**	**85**	**87**	**102**	**110**	**141**	**142**	**143**	**166**
**Wuhan Hu-1**	**G**	**T**	**K**	**P**	**L**	**S**	**L**	**I**	**P**	**P**	**S**	**R**	**G**	**P**	**Q**	**M**	**E**	**E**	**H**	**K**	**S**	**F**	**S**
June 2021 (N=7)	S	I			I	F	F	T	L		P	K	R		H			K					
July 2021 (N=10)	S	I			I	P	F	T	L	R		K	R	L	H	del		K		del	del	del	
August 2021 (N=16)	S	I		S		P	F	T	L	R		K	R	L	H			K					
September 2021 (N=11)	S	I			I	P	F	T	L	R	P	K	R	L	H			K	L				G
October 2021 (N=4)	S		R		I	F	F	T	L	R		K	R	L	H			K					
November 2021 (N=1)	S					F		T	L			K	R		H			K					
December 2021 (N=2)	S	I			I		F	T	L			K	R		H			K					

Genes	nsp2			nsp4				nsp6				nsp10	nsp14	nsp15	nsp16	ORF3a												
**Positions**	**157**	**181**	**429**	**33**	**233**	**289**	**438**	**37**	**106**	**107**	**108**	**111**	**68**	**33**	**160**	**18**	**33**	**34**	**67**	**95**	**155**	**181**	**216**	**223**	**229**	**238**	**255**	**275**
**Wuhan Hu-1**	**V**	**P**	**T**	**M**	**V**	**I**	**L**	**L**	**S**	**G**	**F**	**T**	**G**	**T**	**K**	**G**	**A**	**T**	**K**	**L**	**D**	**E**	**S**	**T**	**T**	**D**	**V**	**L**
June 2021 (N=7)					I		P		del	del	del				R						Y		L		N		del	F
July 2021 (N=10)	I		I				P		del	del	del					V								I	N	Y	del	
August 2021 (N=16)		T	I	C			P		del	del	del								T			A		I	N	Y	del	
September 2021 (N=11)			I				P	F	del	del	del	I					S	M		F				I	N	Y	del	
October 2021 (N=4)			I				P		del	del	del			I									L		N		del	F
November 2021 (N=1)						V	P		del	del	del												L				del	F
December 2021 (N=2)									del	del	del		A										L		N		del	F

Genes	ORF7a	ORF7b	ORF8				Plpro											Rdrp				
**Positions**	**43**	**40**	**7**	**16**	**120**	**121**	**93**	**126**	**153**	**237**	**385**	**428**	**819**	**822**	**864**	**1274**	**1693**	**140**	**184**	**323**	**337**	**744**
**Wuhan Hu-1**	**N**	**T**	**L**	**F**	**F**	**I**	**S**	**S**	**P**	**T**	**I**	**T**	**T**	**P**	**T**	**H**	**K**	**D**	**Q**	**P**	**G**	**E**
June 2021 (N=7)										I		I	I			Y		A		L		D
July 2021 (N=10)		I							S			I	I	S						L		
August 2021 (N=16)	K		S		V	L	F	P			V	I	I	S	A		N			L	S	
September 2021 (N=11)			S	L								I	I	S	A					L		
October 2021 (N=4)			S									I	I							L		
November 2021 (N=1)												I	I						R	L		
December 2021 (N=2)												I	I							L		

Genes	S																														
**Positions**	**9**	**25**	**26**	**67**	**75**	**105**	**136**	**143**	**144**	**152**	**190**	**215**	**241**	**242**	**243**	**244**	**440**	**449**	**477**	**478**	**484**	**501**	**585**	**614**	**655**	**679**	**681**	**716**	**859**	**879**	**1101**
**Wuhan Hu-1**	**P**	**P**	**P**	**A**	**G**	**I**	**C**	**V**	**Y**	**W**	**R**	**D**	**L**	**L**	**A**	**L**	**N**	**Y**	**S**	**T**	**E**	**N**	**L**	**D**	**H**	**N**	**P**	**T**	**T**	**A**	**H**
June 2021 (N=7)	L	L					F		del		S	G			del	del		H			K	Y	F	G	Y	K		I	N		
July 2021 (N=10)	L	L					F	A	del	R	S	G			del	del		H		K	K	Y		G	Y	K	H	I	N	T	Q
August 2021 (N=16)	L	L		V			F	A	del	R	S	G			del	del		H		K	K	Y	F	G	Y	K	H	I	N	T	Q
September 2021 (N=11)	L	L				V	F		del	R	S	G			del	del		H		K	K	Y		G	Y	K		I	N	T	Q
October 2021 (N=4)	L	L	S				F		del		S	G			del	del		H	N	K	K	Y	F	G	Y	K		I	N	T	Q
November 2021 (N=1)	L	L					F		del		S	G	del	del	del			H			K	Y	F	G	Y	K		I	N		
December 2021 (N=2)	L	L					F		del		S	G	del	del	del	del		H			K	Y		G	Y	K		I	N		

*3C-like protease (3CLpro), envelope (E), membrane (M), nucleocapsid (N), non-structural protein (nsp), open-reading frame (ORF), papain-like protease (PLpro), RNA-dependent RNA-polymerase RdRp, spike (S)

Table S11. Mutations detected across the whole genome of C.2 lineage from 2020

Genes	3CLpro	N		nsp2	nsp4	nsp6	nsp14	nsp15	nsp16	ORF3a	ORF7a	PLpro	Rdrp		S			
**Positions**	**15**	**203**	**204**	**274**	**264**	**37**	**85**	**44**	**182**	**23**	**63**	**428**	**302**	**323**	**614**	**623**	**679**	**1219**
**Wuhan Hu-1**	**G**	**R**	**G**	**L**	**L**	**L**	**A**	**E**	**K**	**A**	**F**	**T**	**L**	**P**	**D**	**A**	**N**	**G**
July 2020 (N=1)	S	K	R		I		S					I	S	L	G			V
August 2020 (N=2)	S	K	R	F			S	V			del	I	S	L	G	V	S	
September 2020 (N=3)	S	K	R			F	S		N	S		I	S	L	G			

*3C-like protease (3CLpro), nucleocapsid (N), non-structural protein (nsp), open-reading frame (ORF), papain-like protease (PLpro), RNA-dependent RNA-polymerase RdRp, spike (S)
